# Development and validation of a prognostic nomogram for Takotsubo syndrome patients in the intensive care units: a retrospective cohort study

**DOI:** 10.1038/s41598-022-27224-5

**Published:** 2023-01-10

**Authors:** Jun Chen, Yimin Wang, Xinyang Shou, Qiang Liu, Ziwei Mei

**Affiliations:** 1 Zhejiang Chinese Medical University, Hangzhou, 310000 Zhejiang China; 2 Lishui Municipal Central Hospital, The Fifth Affiliated Hospital of Wenzhou Medical University, Lishui, 323000 Zhejiang China

**Keywords:** Biomarkers, Cardiology, Diseases, Medical research, Risk factors

## Abstract

Patients with Takotsubo syndrome (TTS) admitted to the intensive care unit (ICU) always confront a higher risk of in-hospital death than those hospitalized in the cardiology unit. The prognosis of the latter was analyzed by a large number of studies. However, there was no utility model to predict the risk of in-hospital death for patients with TTS in the ICU. This study aimed to establish a model predicting in-hospital death in patients with TTS admitted to ICU. We retrospectively included ICU patients with TTS from the MIMIC-IV database. The outcome of the nomogram was in-hospital death. Least Absolute Shrinkage Selection Operator (LASSO) analysis selected predictors preliminarily. The model was developed by multivariable logistic regression analysis. Calibration, decision curve analysis (DCA), and receiver operating characteristic (ROC) measured the performance of the nomogram on the accuracy, clinical utility, and discrimination, respectively. Eventually, 368 ICU patients with TTS were enrolled in this research. The in-hospital mortality was 13.04%. LASSO regression and multivariate logistic regression analysis verified risk factors significantly associated with in-hospital mortality. They were potassium, prothrombin time (PT), age, myocardial infarction, white cell count (WBC), hematocrit, anion gap, and sequential organ failure assessment (SOFA) score. This nomogram excellently discriminated against patients with a risk of in-hospital death. The area under curve (AUC) was 0.779 (95%CI: 0.732–0.826) in training set and 0.775 (95%CI: 0.711–0.839) in test set. The calibration plot and DCA showed good clinical benefits for this nomogram. We developed a nomogram that predicts the probability of in-hospital death for ICU patients with TTS. This nomogram was able to discriminate patients with a high risk of in-hospital death and performed clinical utility.

## Introduction

Takotsubo syndrome is a clinical syndrome, viewed as “stimulation cardiomyopathy,” “broken heart syndrome,” or “transient apical ballooning”. It is characterized an acute and transient left ventricular dysfunction. TTS is always induced by physical or emotional stimulation, however, up to 40% of TTS patients present without any trigger event^1^. TTS is easily misdiagnosed as an acute coronary syndrome (ACS), approximately 1–3% of all patients who present symptoms consistent with acute coronary syndrome and undergo coronary angiography have been identified as TTS^2^. Despite numerous studies reporting TTS as a reversible myocardial insufficiency, almost half of the patients with TTS occur cardiovascular complications. Furthermore, the inpatient mortality due to cardiogenic shock, myocardial rupture, or life-threatening arrhythmias is comparable to MI (4–5%)^3–6^. Several serious complications may increase mortality, which is comparable to that of patients with ACS^7^. Recently, the reported mortality rate is higher than the previous^8^. One research based on the data from the SCAAR (Swedish Coronary Angiography and Angioplasty Registry) published that the 30-day mortality rate in TTS was higher than non-STEMI (NSTEMI)^9^.

Compared with non-ICU patients with TTS, ICU patients encounter a higher risk of in-hospital death. One study reported that the mortality occurrence of ICU patients with TTS was much higher than the mortality of patients with TTS admitted to the cardiology unit (30.8% vs. 4.1%)^10^. ICU patients always present multiple organ failures and unstable physical conditions. They have much more risk factors threatening life in the short term than non-ICU patients. Reduction of in-hospital death in ICU patients with TTS plays a vital role in prognosis. However, previous studies mostly involved the investigation of clinical outcomes in non-ICU patients with TTS. Prevention of in-hospital death in ICU patients with TTS hasn’t been addressed and emphasized. For this kind of population, a predictive model effectively identifies how great in-hospital death exposure individuals faced. Clinicians can take action to decrease the risk according to the predicted outcome. Therefore, a prediction model is necessary to develop for decreasing the rate of in-hospital death in ICU patients with TTS.

Nowadays, the clinical prediction model is a good tool that provides valuable guidance for clinical decision-making. Nomogram as a visually predictive model calculates the risk scores for individuals. It is convenient for clinicians to estimate the clinical outcomes of patients. Nomogram has been widely applied in the evaluation of prognosis in critically ill patients these years^11–15^. Therefore, we aimed to develop a nomogram that indicates the probability of in-hospital death for ICU patients with TTS. Clinicians will early discriminate against patients with a higher risk of death by this nomogram and make decisions on treatment as well as intensive care.

## Methods

### Data source

In this study, we retrospectively extracted data from the MIMIC-IV database (version 1.0). The MIMIC-IV database extensively contained all medical records of patients admitted to ICU or the emergency department between 2008–2019 in the Beth Israel Deaconess Medical Center (BIDMC)^16^. The latest version of the MIMIC-IV database is version 1.0. One of the authors (C.J, certification ID: 8,979,131) in this study gained permission to document the database after online training at the National Institutes of Health (NIH). This study relied exclusively on publicly available and anonymous data. As a result, individuals’ permission wasn’t required. To ensure patients' privacy, all methods were carried out by relevant guidelines.

### Patients selection

We enrolled patients who aged more than 18 years old. They were hospitalized in ICU and diagnosed with TTS by the International Classification of Diseases version 9 (“42,983”) and version 10 diagnosis codes (“I5181”) in the MIMIC-IV database. We excluded patients according to the following criteria: (I) No survival outcome data; (II) Being pregnant and the postpartum condition; (III) Incomplete or unobtainable documented or other vital medical records.

### Clinical and laboratory data

All data analyzed in this study included general information, vital signs, and laboratory tests. The time points of vital signs and laboratory parameters used in this analysis were the first document after hospital admission within 24 h. The general information included age, height, weight, and comorbidities such as diabetes, hypertension, chronic lung disease, myocardial infarction, and heart failure. Vital signs contained mean blood pressure (MBP), diastolic blood pressure (DBP), systolic blood pressure (SBP), body temperature (T), respiratory rate (RR), heart rate (HR), pulse oximetry-derived oxygen saturation (SpO_2_). Blood laboratory tests consisted of hemoglobin, hematocrit, creatinine, anion gap, lactate, blood urea nitrogen (BUN), pH, white blood cell count, platelet count, chloride, glucose, prothrombin time (PT), serum potassium, serum sodium, and serum calcium. Therapies were also recorded containing the use of vasoactive drugs (norepinephrine), and continuous renal replacement therapy (CRRT) during the hospitalization. The sequential organ failure assessment (SOFA) score^17^ of every patient also be calculated after hospital admission within 24 h. In this study, the endpoint was in-hospital mortality viewed as survival status at hospital discharge.

### Statistical analysis

The whole dataset was randomly divided into a training set and a test set with a proportion of 7:3. Mean ± standard deviation (SD) documented the normal distribution of continuous variables. Medians with upper and lower quartiles described the abnormal distribution of continuous variables. Continuous variables were tested by T-test or Wilcoxon rank-sum test and categorical variables were analyzed by chi-square test or Fisher's exact test for group comparisons. Firstly, LASSO regression preliminarily screened predictors based on the whole study database. Secondly, these predictors were further analyzed by multivariable regression. Recognized predictors were covered to establish a nomogram based on the training set. The scores for each predictor were calculated based on coefficients of logistic regression variables in the model. A discriminate ability of the nomogram was evaluated by ROC. The AUC of the ROC of more than 0.7 demonstrated a nomogram with discriminate capacity. The calibration curve analyzed by Hosmer–Lemeshow test assessed the fitting degree of nomogram. The DCA evaluated the clinical utility by quantifying net benefits against a range of threshold probabilities. Nomogram was validated in the test set. These results were presented by odds ratio (OR) with 95% confidence intervals (CIs). All tests were two-tailed tests and P ≤ 0.05 was considered statistically significant. We used STATA 15.1 (Stata Corporation, College Station, Texas, USA) and R version 3.6.2 (R Foundation for Statistical Computing, Vienna, Austria) for statistical analysis.

### Ethics approval and consent to participate

The establishment of this database was approved by the Massachusetts Institute of Technology (Cambridge, MA) and Beth Israel Deaconess Medical Center (Boston, MA), and consent was obtained for the original data collection. Therefore, the ethical approval statement and the need for informed consent were waived for this manuscript.

## Results

### The characteristics of study patients

There were 368 eligible patients (85 males and 283 females) in this study. Their average age was 66.29 ± 16.09 years old. 48 patients (16 males and 32 females) died during the hospitalization. The incidence of in-hospital death was 13.04%. Non-survivors tend to be older, and with higher values of anion gap, WBC count, and SOFA score (as shown in Table 1). In the male group, the in-hospital mortality was 18.82% (16/85) and in the female group, the in-hospital mortality was 11.31% (32/283), there was no significant difference between the male group and female group (P = 0.105). Male patients tend to have a worse renal function, as shown in Supplementary Table 1. In addition, Kaplan-Meir survival analysis showed no significant difference in in-hospital mortality between males and females (as shown in Supplementary Fig. 1). The whole sample was randomly divided into a training set and a test set with a proportion of 7:3, and there was no significant difference in variables between the training set and the validation set (as shown in Table 2).

**Table 1:** Baseline characteristics of study patients stratified by survival outcome.

Characteristic	Total (n = 368)	Survival (n = 320)	Death (n = 48)	P value
Age (years old)	66.29 ± 16.09	65.42 ± 16.31	72.09 ± 13.30	**0.007**
Man	85 (23.10%)	69 (21.56%)	16 (33.33%)	0.105
SBP (mmHg)	112.00 ± 15.25	112.43 ± 15.40	109.60 ± 18.36	0.170
DBP (mmHg)	65.70 ± 10.64	62.72 ± 10.86	60.91 ± 12.60	0.913
MBP (mmHg)	78.65 ± 10.43	78.88 ± 10.69	74.80 ± 12.85	0.284
Heart rate (beats/minute)	91.40 ± 17.08	90.40 ± 16.71	89.48 ± 19.66	0.147
Respiratory rate (beats/minute)	21.03 ± 4.12	20.98 ± 4.11	22.06 ± 4.63	0.549
Temperature (°C)	36.89 ± 0.48	36.89 ± 0.48	36.27 ± 1.21	0.518
SPO_2_ (%)	96.95 ± 2.35	96.89 ± 2.43	94.95 ± 7.49	0.231
**Comorbidities, n (%)**				
Diabetes	89 (24.18%)	77 (24.06%)	12 (25.00%)	1.000
Hypertension	157 (42.66%)	141 (44.06%)	16 (33.33%)	0.213
myocardial infarction	111 (30.16%)	102 (31.88%)	9 (18.75%)	0.093
congestive heart failure	212 (57.61%)	187 (58.44%)	25 (52.08%)	0.500
Chronic pulmonary disease	99 (26.90%)	82 (25.63%)	17 (35.42%)	0.211
Malignant cancer	55 (14.95%)	47 (14.69%)	8 (16.67%)	0.887
Renal disease	58 (15.76%)	51 (15.94%)	7 (14.58%)	0.978
**Laboratory parameters**				
Anion gap (mEq/L)	17.63 ± 5.41	15.64 ± 4.02	17.53 ± 4.69	**0.003**
BUN (mg/dL)	33.83 ± 24.64	23.84 ± 18.59	26.35 ± 13.70	0.369
Bicarbonate (mmol/L)	20.80 ± 5.18	22.11 ± 4.52	20.83 ± 5.18	0.075
Creatinine (mg/dL)	1.96 ± 1.98	1.24 ± 1.35	1.16 ± 0.72	0.697
Chloride (mmol/L)	103.83 ± 6.66	103.64 ± 6.48	101.78 ± 7.77	0.072
Glucose (mg/dL)	188.89 ± 94.74	171.85 ± 70.62	203.25 ± 109.05	0.706
Calcium	8.36 ± 1.19	8.22 ± 0.80	8.06 ± 0.89	0.198
Hematocrit (%)	32.70 ± 6.37	33.03 ± 6.29	30.54 ± 6.49	**0.011**
Hemoglobin (g/dL)	10.69 ± 2.15	10.79 ± 2.13	9.98 ± 2.19	**0.015**
WBC (10^9^/L)	13.83 ± 7.74	13.46 ± 7.72	16.27 ± 7.45	**0.019**
Platelet (10^9^/L)	240.37 ± 139.90	239.79 ± 138.51	244.25 ± 150.35	0.837
Potassium (mmol/L)	4.20 ± 0.63	4.17 ± 0.63	4.35 ± 0.61	0.067
PT	15.91 ± 8.13	15.41 ± 6.94	19.22 ± 13.25	**0.002**
Sodium (mmol/L)	138.19 ± 5.25	138.31 ± 5.03	137.40 ± 6.52	0.259
Renal replacement therapy, n (%)	18 (4.89%)	15 (4.69%)	3 (6.25%)	0.913
norepinephrine, n (%)	147 (39.95%)	126 (39.38%)	21 (43.75%)	0.675
**Scoring systems**				
SOFA	5.98 ± 4.00	5.78 ± 3.87	7.27 ± 4.64	**0.016**
ICU LOS, days	5.82 ± 6.75	5.85 ± 9.19	5.56 ± 6,17	0.782
HOS LOS (days)	17.75 ± 17.91	18.51 ± 18.34	14.20 ± 14.30	0.120

SBP: systolic blood pressure; DBP: diastolic blood pressure; MBP: mean blood pressure; SPO_2_: pulse oximetry derived oxygen saturation; BUN: blood urea nitrogen; PT: prothrombin time; WBC: white blood cell; SOFA: sequential organ failure assessment; ICU: intensive care unit; HOS: hospital; LOS: length of stay.

Significant values are in bold.

**Table 2:** The characteristic of training dataset and test dataset subjects.

Characteristic	Total (n = 368)	Training set (n = 258)	Test set (n = 110)	P value
Age (years old)	66.29 ± 16.09	66.29 ± 16.31	66.27 ± 15.63	0.988
Man	85 (23.10%)	55 (21.31%)	30 (27.30%)	0.269
SBP (mmHg)	112.00 ± 15.25	111.72 ± 15.35	112.67 ± 15.07	0.583
DBP (mmHg)	65.70 ± 10.64	65.75 ± 10.60	65.56 ± 10.80	0.874
MBP (mmHg)	78.65 ± 10.43	78.38 ± 10.39	79.29 ± 10.53	0.448
Heart rate (beats/minute)	91.40 ± 17.08	91.94 ± 16.59	90.12 ± 18.20	0.348
Respiratory rate (beats/minute)	21.03 ± 4.12	21.10 ± 4.24	20.87 ± 3.83	0.627
Temperature (°C)	36.89 ± 0.48	36.88 ± 0.50	36.93 ± 0.41	0.314
SPO_2_ (%)	96.95 ± 2.35	96.91 ± 2.14	97.04 ± 2.78	0.645
**Comorbidities, n (%)**				
Diabetes	89 (24.18%)	58 (22.48%)	31 (28.19%)	0.300
Hypertension	157 (42.66%)	112 (43.41%)	45 (40.91%)	0.410
myocardial infarction	111 (30.16%)	75 (29.07%)	36 (32.73%)	0.773
congestive heart failure	212 (57.61%)	147 (56.98%)	65 (59.10%)	0.794
Chronic pulmonary disease	99 (26.90%)	73 (28.29%)	26 (23.64%)	0.427
Malignant cancer	55 (14.95%)	41 (15.89%)	14 (12.73%)	0.603
Renal disease	58 (15.76%)	46 (17.83%)	12 (10.91%)	0.131
**Laboratory parameters**				
anion gap (mEq/L)	17.63 ± 5.41	15.75 ± 4.05	16.20 ± 4.40	0.342
BUN (mg/dL)	33.83 ± 24.64	24.47 ± 18.81	23.47 ± 16.12	0.630
Bicarbonate (mmol/L)	20.80 ± 5.18	21.99 ± 4.43	21.84 ± 5.07	0.776
Creatinine (mg/dL)	1.96 ± 1.98	1.24 ± 1.17	1.20 ± 1.51	0.799
Chloride (mmol/L)	103.83 ± 6.66	103.29 ± 6.68	103.64 ± 6.70	0.652
Glucose (mg/dL)	188.89 ± 94.74	149.41 ± 53.65	203.25 ± 109.05	0.384
calcium	8.36 ± 1.19	8.18 ± 0.83	8.26 ± 0.78	0.358
Hematocrit (%)	32.70 ± 6.37	33.33 ± 6.27	33.58 ± 6.52	0.084
Hemoglobin (g/dL)	10.69 ± 2.15	10.58 ± 2.14	10.92 ± 2.16	0.159
WBC (10^9^/L)	13.83 ± 7.74	13.87 ± 7.49	13.73 ± 8.33	0.871
Platelet (10^9^/L)	240.37 ± 139.90	243.91 ± 144.80	232.05 ± 127.92	0.457
Potassium (mmol/L)	4.20 ± 0.63	4.19 ± 0.65	4.21 ± 0.59	0.801
PT	15.91 ± 8.13	15.56 ± 6.72	16.72 ± 10.71	0.210
Sodium (mmol/L)	138.19 ± 5.25	138.00 ± 5.43	138.64 ± 4.80	0.287
Renal replacement therapy, n (%)	18 (4.89%)	14 (5.43%)	4 (3.64%)	0.642
Norepinephrine, n (%)	147 (39.95%)	104 (40.31%)	43 (39.10%)	0.918
**Scoring systems**				
SOFA	5.98 ± 4.00	6.07 ± 4.07	5.78 ± 3.86	0.521
ICU LOS, days	5.82 ± 6.75	5.85 ± 6.78	5.80 ± 6.83	0.944
HOS LOS (days)	17.75 ± 17.91	17.68 ± 17.96	17.68 ± 18.09	0.885

SBP: systolic blood pressure; DBP: diastolic blood pressure; MBP: mean blood pressure; SPO_2_: pulse oximetry derived oxygen saturation; BUN: blood urea nitrogen; PT: prothrombin time; WBC: white blood cell; SOFA: sequential organ failure assessment; ICU: intensive care unit; HOS: hospital; LOS: length of stay.

### Predictors and nomogram for in-hospital mortality

**Figure 1:**
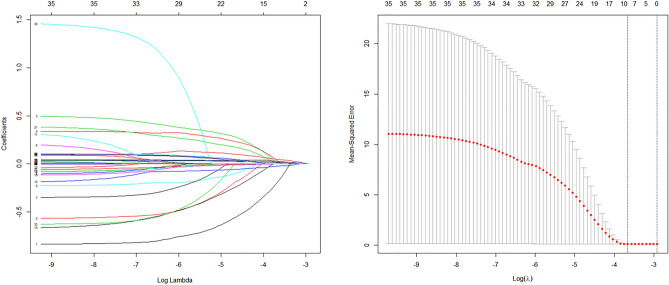
Texture feature selection using the least absolute shrinkage and selection operator (LASSO) binary logistic regression model. (**a**) Each curve in the figure represents the change trajectory of each independent variable coefficient. The ordinate is the value of the coefficient, the lower abscissa is log(λ), and the upper abscissa is the number of non-zero coefficients in the model at this time. **(b)** tenfold cross-cross validation fitting and then selecting the model, and at the same time have a more accurate estimate of the performance of the model. For each λ value, around the mean value of the target parameter shown by the red dot, we can obtain a confidence interval for the target parameter. The two dashed lines indicate two special λ values:c (cvfit$lambda.min, cvfit$lambda.1se). The mean squared error was plotted vs. log(λ).

**Figure 2:**
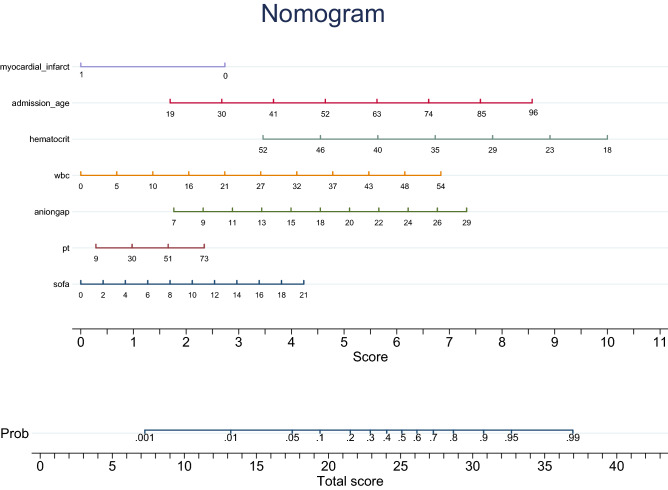
The nomogram for predicting the risk of in-hospital mortality in takotsubo syndrome patients. The top row of the ‘Points’ represents a scale for each risk factors, and points of each predictor were acquired by drawing a straight line upwards from the corresponding value to the “Points” line. Then, the points received from each predictor are summed, and the number is located on the “Total Points” axis. To conclude the patient’s sort of probability for in-hospital mortality, draw a straight line down to the corresponding “Risk of death” axis. resp_rate: respiratory rate. For example, a 74-years-old male without myocardial infarction history, his clinical data of admission was as followed: PT: 30 s, anion gap: 24 mEq/L, WBC: 10*10^9, hematocrit: 35% and SOFA score were 4 points. The corresponding score of each predictor were 6.8 points, 2.5 points, 1.0 points, 6.2 points, 1.3 points, 6.7 points, and 0.8 points respectively. Then his total score was about 24.5 points, and the risk of in-hospital mortality is 45.5%.

We performed lasso regression analysis to preliminarily select predictors (as shown in Fig. 1). Screened variables had a large regression coefficient meaning great influence on the outcomes. They were age, myocardial infarction history, gender, potassium, PT, WBC, hematocrit, anion gap, and SOFA score. Finally, age, myocardial infarction history, WBC, anion gap, PT, and SOFA score were demonstrated as independent risk factors for in-hospital mortality of ICU patients with TTS (as shown in Table 3). We included the above independent risk factors in the nomogram (as shown in Fig. 2).

**Table 3:** Lasso logistic regression and multivariate logistic regression analysis of the risk factors for in-hospital death in TTS.

Variable	Lasso regression analysis		Multivariable regression analysis	
	OR (95% CI)	P value	OR (95% CI)	P value
Age (years old)	**1.003 (1.001–1.005)**	**0.024**	**1.003 (1.001–1.005)**	**0.016**
Gender (man)	1.078 (0.994–1.170)	0.072	1.051 (0.970–1.139)	0.104
myocardial infarction	**0.924 (0.870–0.988)**	**0.041**	**0.920 (0.864–0.991)**	**0.043**
Hematocrit	**0.991 (0.985–0.999)**	**0.009**	**0.988 (0.993–0.999)**	**0.011**
WBC	**1.008 (1.003–1.014)**	**0.005**	**1.004 (1.000–1.009)**	**0.032**
anion gap	**1.014 (1.004–1.024)**	**0.008**	**1.011 (1.002–1.019)**	**0.026**
Potassium	1.039 (0.975–1.108)	0.238	1.025 (0.971–1.083)	0.876
PT	1.003 (0.997–1.009)	0.121	**1.005 (1.001–1.009)**	**0.045**
SOFA	**1.011 (1.001–1.021)**	**0.038**	**1.010 (1.001–1.017)**	**0.047**

WBC: white blood cell; PT: prothrombin time; SOFA: sequential organ failure assessment.

Significant values are in bold.

### Evaluation and validation of the nomogram

**Figure 3:**
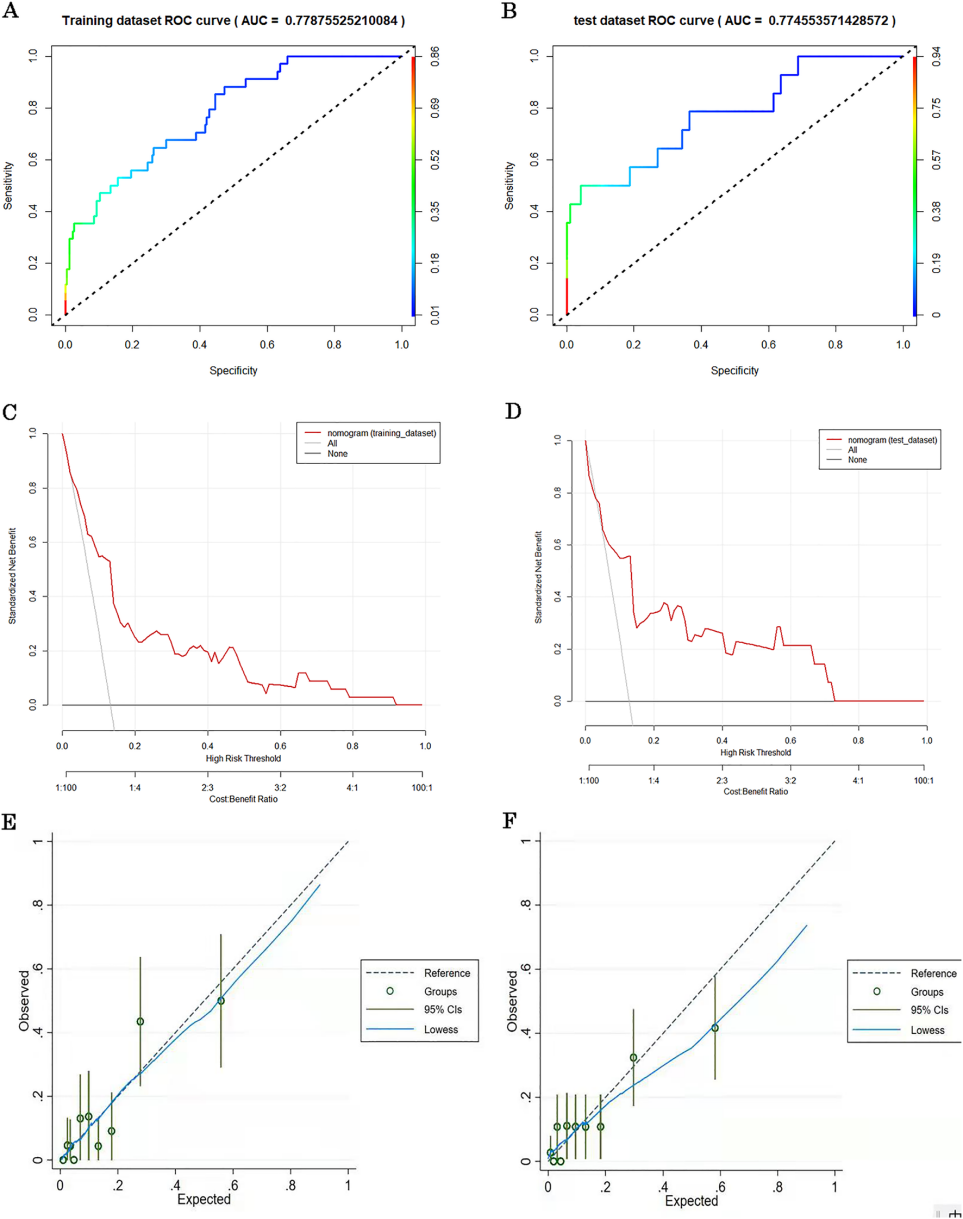
(**A**) Receiver operating characteristic (ROC) curve of the nomogram in the training set. The area under the curve (AUC) of ROC was (AUC: 0.779, 95% CI: 0.732–0.826). (**B**) Receiver operating characteristic (ROC) curve of the nomogram in the test set. The area under the curve (AUC) of ROC was (AUC: 0.775, 95% CI: 0.711–0.839). (**C**) Calibration curve of the nomogram in the training set. The x-axis represents the predicted probability of in-hospital mortality of TTS patients. The y-axis represents the actual in-hospital mortality of TTS patients. The diagonal dotted line represents a perfect prediction by an ideal model. The solid line represents the performance of the nomogram, of which a closer fit to the diagonal dotted line represents a better prediction. The figure shows that the prediction model have a good predictive ability. (**D**) Calibration curve of the nomogram in the test set. The x-axis represents the predicted probability of in-hospital mortality of TTS patients. The y-axis represents the actual in-hospital mortality of TTS patients. The diagonal dotted line represents a perfect prediction by an ideal model. The solid line represents the performance of the nomogram, of which a closer fit to the diagonal dotted line represents a better prediction. The figure shows that the prediction model has a good predictive ability. (**E**) Decision curve analysis of the nomogram for TTS patients (training set). The DCA curve of the nomogram for TTS patients. Solid line: The patient does not apply the nomogram, and the net income is zero; Gray line: All patients used the nomogram. The further the red solid line was from the dotted line, the greater the clinical application value. (**F**) Validation of the DCA curve of the nomogram for TTS patients (test set). Solid line: The patient does not apply the nomogram, and the net income is zero; Gray line: All patients used the nomogram. The further the red solid line was from the dotted line, the greater the clinical application value.

This prediction model showed a good discriminate ability (AUC: 0.779, 95% CI: 0.732–0.826) (Fig. 3A). We validated the prediction model in the test set. A similar ROC result was found (AUC: 0.775, 95% CI: 0.711–0.839) (Fig. 3B). Additionally, we performed calibration curve analysis to test the fitting degree of the nomogram. The calibration curve plot indicated a concordance between predicted probabilities and observed death rates both in the test and training sets (as shown in Fig. 3C and Fig. 3D). We evaluated the clinical utility of the prediction model by decision curve analysis. DCA curve demonstrated a clinical utility in model practice (as shown in Fig. 3E and F).

## Discussion

In recent years, various studies have analyzed the prognosis of patients with TTS^18–22^, however, they mostly focused on the long-term prognosis. There was still limited research on the short-term prognosis, especially for ICU patients with TTS. These patients have a higher rate of mortality than non-ICU patients with TTS^10^. This study retrospectively investigated ICU patients with TTS. The incidence of in-hospital mortality was 13.04%. The rate was higher than previously reported mortality of non-ICU patients (2–5%)^3–6^. Compared with non-ICU patients, a majority of ICU patients are associated with multiple organ failures, hemodynamic instability, and severe complications. They often suffer from TTS triggered by physical stress. These risk factors lead them to be more serious and complicated. The probability of in-hospital death always is higher than in non-ICU populations. In this nomogram, age, myocardial infarction history, PT, WBC, hematocrit, anion gap, and SOFA score were covered as predictors for in-hospital mortality of TTS patients. This nomogram presented a good predictive ability.

Age and SOFA score were viewed as risk factors for in-hospital death in ICU patients with TTS. The mean age of non-survivors tended to be older than survivors (72.09 ± 13.30 vs 65.42 ± 16.31, P = 0.007). A previous report divided participants according to age. They showed the generation of 50–64 years (OR = 0.75, P = 0.05) as a predictor for in-hospital mortality^19^. Different from this report, this nomogram found a risk of death in patients aging of 19–96 years to a different extent. Each additional 11 years significantly increases the risk by 9%. Patients (> 68 years) are exposed in death risk of more than 50%. SOFA score evaluated the severity of illness by describing the time course of multiple organ dysfunction^23^. Previous studies supposed SOFA score as a predictor of clinical outcome^24^. In this study, the SOFA score of non-survivors was statistically higher than survivors (7.27 vs 5.78, P = 0.016). This nomogram indicates a risk of in-hospital mortality when the SOFA score is more than five.

Santoro et al. stratified the risk of in-hospital complications in patients with TTS by a GEIST score^25^. The GEIST score doesn’t consist of laboratory values. However, we discovered an association between in-hospital death with PT, WBC, and anion gap. PT is used to estimate the tissue factor and coagulation pathways. A prolonged PT indicates a defect of the coagulation factor or the presence of coagulation factor inhibitors^26^. Patients with PT prolongation always have a higher risk of hemorrhage. Previous studies discovered that aneurysmal subarachnoid hemorrhage (aSAH) can trigger TTS. TTS patients with aSAH have a higher risk of in-hospital mortality.^27,28^. When patients with TTS occur a prolongation of PT, clinicians should evaluate the risk of hemorrhage and monitor the adverse reaction created by anticoagulant. Anion gap is applied to evaluate the acid–base equilibrium. Anion gap elevation results from acute or chronic acid–base disorders, such as lactic acidosis, ketoacidosis, toxic alcohol poisoning, salicylate intoxication, and acute or chronic kidney disease^29^. Some cases reported acidosis as one of the trigger reasons for TTS, especially ketoacidosis^30^. Acidosis physiologically increases stress hormones such as catecholamines, and cortisol and deleteriously affect myocyte. In this nomogram, the in-hospital mortality risk is less than 5% in patients with an anion gap of 8–16 mmol/L. When the anion gap is more than 18 mmoL/L, every 2 mmoL/L in elevation of anion gap concentration increases mortality risk by 10–20%. White cell count is an important value in the prediction of TTS severity, they participate in process of oxidative stress. These three values haven’t been discussed in previous reports, we first found their impact on in-hospital death for ICU patients with TTS.

In this study, higher hematocrit and history of infarction history reduce the risk of in-hospital mortality. Hematocrit is one of laboratory tests used to diagnose anemia. Patients with lower hematocrit levels tend to be anemia. Some evidence supported the relationship of anemia with poor outcomes of TTS^31,32^. Some analyses applied hematocrit to predict in-hospital death in patients with heart failure. They demonstrated a mortality incidence of more than 50% in heart failure patients with a hematocrit of less than 30%^33^. The normal hematocrit levels are 40%-50% in males and 37–48% in females. This nomogram for the first time predicted a higher mortality risk of more than 16% in TTS patients (hematocrit < 37%), compared with patients (hematocrit 37–52%). There is limited evidence on the role of myocardial infarction history on in-hospital death of TTS. In this study, survivors combined with myocardial infarction were higher than non-survivors (31.88% vs 18.75%, P = 0.093). The protective role of myocardial infarction history on in-hospital outcomes for TTS patients deserved to investigate in the following research.

Nowadays, it is controversy about whether gender is a risk factor for in-hospital death of TTS. Although female was identified as a risk factor for TTS, researchers haven’t reached a consensus on the predicted effect of female on in-hospital death. This nomogram didn’t include gender as a predictor. We analyzed the baseline characteristics of participants by stratification of gender. We didn’t discover a significant difference between males and females in baseline characteristics, except for BUN, creatinine, and potassium. This finding didn’t support the predicted value of gender on in-hospital mortality. Therefore, gender can’t be a predictor for in-hospital death in ICU patients with TTS.

### Study limitation

Several limitations must be acknowledged. Firstly, we aimed to establish a rapid and simple nomogram for predicting in-hospital mortality in TTS patients. The situation of TTS patients was very complex and many factors are related to the prognosis of their hospitalization. We only included the clinical data of the patients within 24 h after hospital admission and did not consider the intervention measures at the onset or after hospitalization. Secondly, this study did not further analyze the causes of TTS, such as physical triggers or psychological triggers. Future studies can conduct a subgroup analysis of specific causes of cardiac arrest to provide more evidence. Thirdly, some records weren’t included, such as mechanical ventilation, echocardiography parameters, mechanical hemodynamic support record, and intraaortic balloon pumps. Because these records are incomplete. The results of this study were only suitable for ICU patients with TTS.

## Conclusion

We have established a risk prediction model by admission characteristics of ICU patients with TTS. This model provides some evidence to identify patients with a high risk of in-hospital death.

## Supplementary Information

Supplementary Legends.

Supplementary Figure 1.

Supplementary Table 1.

## Data Availability

The datasets generated and analyzed during the current study are available from the corresponding author upon reasonable request.

## Abbreviations

TTS: Takotsubo syndrome
ICD: International Classification of Diseases
AUC: Area under the curve
ROC: Receiver operating characteristic
CI: Confidence intervals
SBP: Systolic blood pressure
DBP: Diastolic blood pressure
MBP: Mean blood pressure
SPO_2_: Pulse oximetry derived oxygen saturation
BUN: Blood urea nitrogen
PT: Prothrombin time
WBC: White blood cell
CRRT: Continuous renal replacement therapy
SOFA: Sequential organ failure assessment
ICU: Intensive care unit
HOS: Hospital
LOS: Length of stay
