# Supplementary material for: Development and validation of a prognostic nomogram for Takotsubo syndrome patients in the intensive care units: a retrospective cohort study
Source: Sci Rep. 2023 Jan 10;13:477. doi: 10.1038/s41598-022-27224-5 (PMC9832151; doi:10.1038/s41598-022-27224-5)
Supplement: Supplementary file 1 — Supplementary Legends. [file 41598_2022_27224_MOESM1_ESM.docx]

**FIGURE LEGENDS**

**Supplementary Figure 1:** The survival curve for patients with different gender was shown in a Kaplan-Meier analysis plot in Supplementary Figure 1. The result showed that the man group had lower survival possibility during the hospitalization than the woman group, but not reached statistical differences (log-rank test: P=0.1).
