# Supplementary figures and images for: Development and validation of a prognostic nomogram for Takotsubo syndrome patients in the intensive care units: a retrospective cohort study
Source: Sci Rep. 2023 Jan 10;13:477. doi: 10.1038/s41598-022-27224-5 (PMC9832151; doi:10.1038/s41598-022-27224-5)

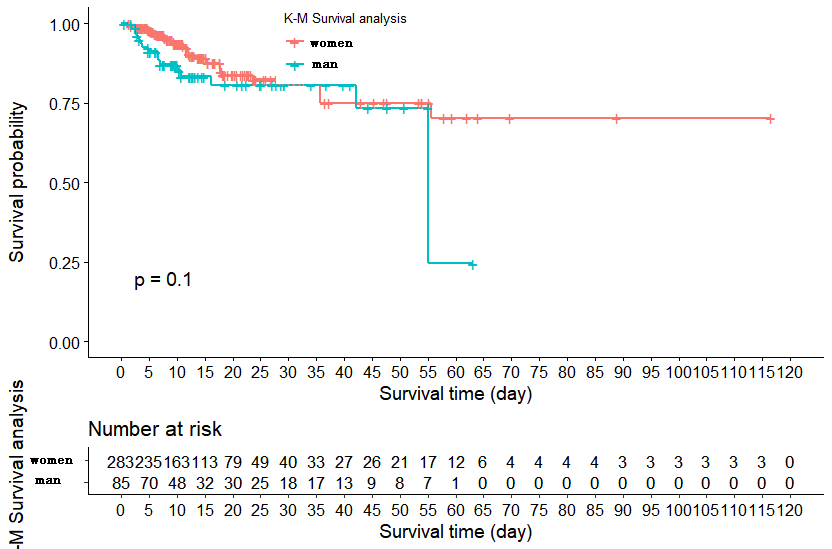

Supplement: Supplementary file 2 — Supplementary Figure 1. [file 41598_2022_27224_MOESM2_ESM.png]
