# Supplementary material for: Development and validation of a prognostic nomogram for Takotsubo syndrome patients in the intensive care units: a retrospective cohort study
Source: Sci Rep. 2023 Jan 10;13:477. doi: 10.1038/s41598-022-27224-5 (PMC9832151; doi:10.1038/s41598-022-27224-5)
Supplement: Supplementary file 3 — Supplementary Table 1. [file 41598_2022_27224_MOESM3_ESM.docx]

**Table 1 Baseline characteristics of study patients stratified by gender**

| Characteristic | Total (n=368) | Women (n=283) | Man (n=85) | P value |
| --- | --- | --- | --- | --- |
| Age (years old) | 66.29±16.09 | 66.73±15.62 | 64.82±17.60 | 0.338 |
| SBP (mmHg) | 112.00±15.25 | 112.34±15.22 | 110.88±15.39 | 0.439 |
| DBP (mmHg) | 65.70±10.64 | 65.86±10.42 | 65.15±11.40 | 0.588 |
| MBP (mmHg) | 78.65±10.43 | 78.83±10.36 | 78.08±10.69 | 0.564 |
| Heart rate (beats/minute) | 91.40±17.08 | 91.58±17.29 | 90.80±16.45 | 0.713 |
| Respiratory rate (beats/minute) | 21.03±4.12 | 21.21±4.17 | 20.44±3.91 | 0.129 |
| Temperature (°C) | 36.89±0.48 | 36.89±0.48 | 36.86±0.47 | 0.417 |
| SPO2 (%) | 96.95±2.35 | 96.91±2.48 | 97.08±1.84 | 0.552 |
| Comorbidities, n (%) |  |  |  |  |
| Diabetes | 89 (24.18%) | 67 (23.70%) | 22 (25.90%) | 0.785 |
| Hypertension | 157 (42.66%) | 114 (40.30%) | 43 (50.60%) | 0.119 |
| myocardial infarction | 111 (30.16%) | 90 (31.80%) | 21 (24.70%) | 0.265 |
| congestive heart failure | 212 (57.61%) | 161 (56.90%) | 51 (60.00%) | 0.701 |
| Chronic pulmonary disease | 99 (26.90%) | 82 (29.00%) | 17 (20.00%) | 0.134 |
| Malignant cancer | 55 (14.95%) | 39 (13.80%) | 16 (18.80%) | 0.332 |
| Renal disease | 58 (15.76%) | 43 (15.20%) | 15 (17.60%) | 0.708 |
| Laboratory parameters |  |  |  |  |
| anion gap (mEq/L) | 17.63±5.41 | 15.72±4.06 | 16.44±4.43 | 0.164 |
| BUN (mg/dL) | 33.83±24.64 | 22.06±15.56 | 31.29±23.18 | **<0.001** |
| Bicarbonate (mmol/L) | 20.80±5.18 | 22.10±4.57 | 21.43±4.80 | 0.245 |
| Creatinine (mg/dL) | 1.96±1.98 | 1.13±1.04 | 1.54±1.84 | **0.010** |
| Chloride (mmol/L) | 103.83±6.66 | 103.72±6.65 | 102.33±6.70 | 0.093 |
| Glucose (mg/dL) | 188.89±94.74 | 171.85±70.62 | 203.25±109.05 | 0.706 |
| calcium | 8.36±1.19 | 8.15±1.11 | 8.20±0.60 | 0.679 |
| Hematocrit (%) | 32.70±6.37 | 32.79±6.17 | 32.40±7.00 | 0.623 |
| Hemoglobin (g/dL) | 10.69±2.15 | 10.71±2.06 | 10.60±2.44 | 0.681 |
| WBC (10^9^/L) | 13.83±7.74 | 13.74±7.62 | 14.11±8.16 | 0.705 |
| Platelet (109/L) | 240.37±139.90 | 246.61±144.65 | 219.58±121.25 | 0.118 |
| Potassium (mmol/L) | 4.20±0.63 | 4.11±0.57 | 4.48±0.75 | **<0.001** |
| PT | 15.91±8.13 | 15.53±7.62 | 16.91±9.59 | 0.169 |
| Sodium (mmol/L) | 138.19±5.25 | 138.39±5.01 | 137.53±5.97 | 0.183 |
| Renal replacement therapy, n (%) | 18 (4.89%) | 12 (4.20%) | 6 (7.10%) | 0.441 |
| norepinephrine, n (%) | 147 (39.95%) | 113 (39.90%) | 34 (40.00%) | 1.000 |
| Scoring systems |  |  |  |  |
| SOFA | 5.98±4.00 | 6.02±4.07 | 5.82±3.81 | 0.685 |
| ICU LOS, days | 5.82±6.75 | 5.89±6.96 | 5.57±6,00 | 0.702 |
| HOS LOS (days) | 17.75±17.91 | 17.67±18.28 | 18.86±16.68 | 0.592 |
| HOS mortality, n (%) | 48 (13.04%) | 32 (11.31%) | 16 (18.82%%) | 0.105 |

SBP: systolic blood pressure; DBP: diastolic blood pressure; MBP: mean blood pressure; SPO2: pulse oximetry derived oxygen saturation; BUN: blood urea nitrogen; PT: prothrombin time; WBC: white blood cell; SOFA: sequential organ failure assessment; APSII: acute physiology score II; ICU: intensive care unit; HOS: hospital; LOS: length of stay.
